# War Pensions Administration

**Published:** 1921-07-16

**Authors:** 


### WAR PENSIONS ADMINISTRATION.

The debates in the House of Commons during the past week on the Bill now under consideration for altering much of the existing system of War Pensions Administration touch only one aspect of the important changes which are occurring in the Ministry of Pensions. The centralisation at which the Bill aims, the removal of authority from amateur local Committees into the hands of paid officials, and most of the other changes apparent in the text of the Bill, are matters as to which two opinions are obviously possible. All that need be said here is that provided always the permanent official is just, really expert, and can free himself from the besetting bureaucratic sin of "eye-wash," his administration is likely to be both more economical and more truly in the interests of the pensioned than that of the well-meaning but too frequently incapable local Committee has been. If, on the other hand, the paid official entrenches himself in soulless regulations, and wires himself in with red-tape, the last state of the disabled will be worse than the first.

On these matters, as has been said, there is room for diversity of opinions; but on the subject of other changes now introduced in the Ministry, but not laid before Parliament, it will be hard not to agree that improvements are being made. An enormous saving of public money will certainly be effected, without any injustice whatever to the disabled. The new reforms have long been overdue, and it is satisfactory that at last serious steps are being taken to stop a leakage that had become a grave public scandal. It should be understood that in what follows it is almost solely those pensioned on account of illness contracted or alleged to have been contracted on active service that are affected. The wounded disabled present a much less difficult problem; in nineteen cases out of twenty of those whose pensions fluctuate from time to time, it is illness, not wounds, that constitutes the disability.

To explain the working of these new amendments it is first necessary to understand that the rate of each man's pension is decided (by a medical board) in terms which are a percentage of the maximum amount, or total disability. To safeguard the interests of the Service man there is an appeal board, to which he can have his case referred if he is dissatisfied with the original assessment. Between these two boards, both of which are animated by the friendliest and most sympathetic feelings towards every genuine case of hardship or disability, and are further directly instructed from the Ministry always to allow the pensioner the benefit of any doubt, it is safe to say that under-assessment is practically unknown; and, in fact, it is notorious to those conversant with the inner workings of the Ministry that these boards over-assess disability quite a hundred times for every time they under-assess it.

Then there has been a regulation—and it is this which is now being repealed, or at least materially revised—that any pensioner in receipt of a pension is at once raised to full rates (technically known as "treatment allowances") wherever a district medical referee certifies that he is unfit for work by reason of any of the conditions for which he is pensioned. Many medical referees interpret this clause very widely, and certify a man for treatment allowances (sometimes for months or years at a time) on very inadequate grounds; often, indeed, whenever a man falls ill, no matter whether his illness has or has not anything to do with his pensionable disability. The Ministry's intention has been that a man shall only be certified unfit for work if he cannot do such proportion of a day's work as that for which he is not pensioned; that is, that a man assessed at 30 per cent. disability shall only be certified unfit for work if he is found to have less than 70 per cent. of an ordinary man's working capacity. Here, again, many medical referees have disregarded the regulations, and put men upon treatment allowances for very insufficient reasons; also there has been, naturally enough, a good deal of malingering amongst pensioners who see a chance of exchanging a 15 per cent. pension, for example, for a 100 per cent. one by the simple process of humbugging a complaisant referee. It may be said that the Ministry cannot be held responsible for the frailty of medical referees; but this argument will not really hold water, for no attempt, or very little, is seriously made to supervise the work of the medical referees or to encourage them in suppressing malingering.

### The New Amendments.

The administrative change which is now introduced, and will, if properly worked, cut down the enormous expenditure on unjustifiable and demoralising pensions, is chiefly contained in a clause which makes it necessary before the treatment allowances can be granted for a medical referee to certify not merely that a man is unable to work by reason of his disability, but also that the treatment necessary renders it in any case impossible for him to work. The necessity for this somewhat cumbrous clause is evidently the failure of a previous instruction to the effect that men put temporarily in charge of a panel practitioner were not in ordinary circumstances to be put instantaneously on "treatment allowances"; but only when the illness was of so serious a character as to require hospital treatment. The result of this was that many medical referees sent every case of sickness in a pensioned Service man to a hospital clinic, although the trouble might be some simple ailment well within the competence of a panel practitioner. The hospital clinics established for serious cases thus became swamped by large numbers of men who should never have been sent to them; the men, of course, being willing enough because they realised that "treatment allowances" followed automatically.

Thus it has happened that a man discharged with some slight ailment such as a tendency to bronchitis or to osteo-arthritis, in many cases pre-existing long before his entry into the Forces, and in many cases,

(Continued on p. 266.)

### WAR PENSIONS ADMINISTRATION.—(*Continued from p. 263.*)

indeed, actually the cause of the man not being sent out of England, has drawn a percentage pension for, say, forty-eight weeks of the year (during which he was in sound health and full capacity for work) and a full pension (" treatment allowances ") for the other four weeks during which an attack of his complaint may have occurred. Another side of the same problem is seen in the large numbers of elderly men, enlisted for the Defence Force only, and never exposed to any serious climatic exposure or to foreign service conditions, who manage to draw " treatment allowances " for months and years at a time for conditions which are most accurately described as old age. These are the cases (and the public at large has no idea what a large number of them there are) that local Committees lack the courage to deal with honestly in many instances. It is the types just illustrated that in the interests of their own self-reliance as well as of the public purse must be dealt with more firmly than hitherto; and we are glad to support the Ministry in any reforms which will achieve this end. The Ministry must realise, however, that the malingering evil will only be overcome when district referees are closely supervised and the weak ones weeded out.

